# Identification and Classification of Lungs Focal Opacity Using CNN Segmentation and Optimal Feature Selection

**DOI:** 10.1155/2023/6357252

**Published:** 2023-07-26

**Authors:** Muhammad Ashar Javed, Hannan Bin Liaqat, Talha Meraj, Aziz Alotaibi, Majid Alshammari

**Affiliations:** ^1^Department of Information Technology, University of Gujrat, Gujrat, Pakistan; ^2^Department of Information Technology, Division of Science and Technology University of Education, Township Campus Lahore, Lahore, Pakistan; ^3^Department of Computer Science, COMSATS University Islamabad—Wah Campus, Wah Cantt, Rawalpindi 47040, Pakistan; ^4^Department of Computer Science, College of Computers and Information Technology, Taif University, P.O. Box 11099, Taif 21944, Saudi Arabia; ^5^Department of Information Technology, College of Computers and Information Technology, Taif University, P.O. Box 11099, Taif 21944, Saudi Arabia

## Abstract

Lung cancer is one of the deadliest cancers around the world, with high mortality rate in comparison to other cancers. A lung cancer patient's survival probability in late stages is very low. However, if it can be detected early, the patient survival rate can be improved. Diagnosing lung cancer early is a complicated task due to having the visual similarity of lungs nodules with trachea, vessels, and other surrounding tissues that leads toward misclassification of lung nodules. Therefore, correct identification and classification of nodules is required. Previous studies have used noisy features, which makes results comprising. A predictive model has been proposed to accurately detect and classify the lung nodules to address this problem. In the proposed framework, at first, the semantic segmentation was performed to identify the nodules in images in the Lungs image database consortium (LIDC) dataset. Optimal features for classification include histogram oriented gradients (HOGs), local binary patterns (LBPs), and geometric features are extracted after segmentation of nodules. The results shown that support vector machines performed better in identifying the nodules than other classifiers, achieving the highest accuracy of 97.8% with sensitivity of 100%, specificity of 93%, and false positive rate of 6.7%.

## 1. Introduction

Cancer professes a great threat worldwide to human health. Among all other types of cancers, lung cancer has the highest death rate. According to the world health report, about 8.2 million deaths occur per year due to cancer and 1.69 million of which are due to lung cancer [1]. The survival rate in lung cancer is very low than other cancers. In spite of the advancement in medical treatments of lungs cancer, its five-year survival rate still fluctuates from 4% to 17%, but if the lung cancer is identified at its early stages, the survival rate can be improved [2]. The key is to identify the exact location of nodules. The lung malignancy is caused by abnormal growth of cells in lungs tissues. Risk factors that cause the cancer to happen are biological reactions, chemical reactions, and smoking. [3].

The human lungs are pyramid in shape paired organs (left and right lungs) that are connected through trachea known as a windpipe. The trachea is further connected to two bronchi (airway in respiratory systems) that holds both the paired organs together and regulates oxygen. There are mainly two bronchi: the one is called left bronchus and the other is called right bronchus; these bronchi are further divided into the secondary and tertiary bronchus. Each lung is further categorized into smaller regions called lobes. The left lung has three smaller sections (lobes) called superior lobe, middle lobe, and inferior lobe while the right lung mainly composes of two lobes called superior and inferior lobes. There may exist some nodules in the lobe's region of lungs [4]. The nodules are the abnormal or irregular growth or a spot in the lobe's region. These nodules that appear in lungs are of two types either they can be benign or malignant. The benign nodules are considered as normal ones while on the other hand, the malignant nodules are threatening which may be the cause of lung cancer.

The lung's nodules are of different sizes. The nodules on the basis of their size are named as micronodules, focal opacity, and mass. The “micro” nodules are the nodules having the distance across under 3 mm, while the nodule having the size ranges from 3 mm to 30 mm is called “focal opacity,” and the nodules with size more than 30 mm are known as “mass” nodules [5].

Nodules can develop in internal tissues; the reason for inflammation may be due to autoimmune reaction or infection. Not all the nodules found in the lobe's region are cancerous and usually do not need cancer treatment, but the nodules bigger then 1.2 inches are more likely to be malignant. There are some difficulties that are associated with the detection of nodules. These nodules are adjacent to the vessels in the lobe regions the bronchi and vessels are having the same intensity and shape like nodules due to this similarity in shape and intensity the radiologists found it difficult to detect the nodule [6].

There are various imaging modalities available in order to detect the nodules. These imaging modalities include computed tomography (CT), positron emission tomography (PET), and magnetic resonance imaging (MRI) [7]. In computerized X-ray imaging, a narrow beam of laser is placed on the patient body which quickly rotates around the body. Signals are produced that are being processed by machine to generate slices or cross-sectional images. CT scan uses multiple detectors that within no time scan whole chest. Positron emission tomography (PET) is an imaging technique in which radioactive tracers are being used that are inserted in to the body (inhaled, injected, or swallowed) to generate images of tissues inside the body in order to examine metabolic processes. This method sometime detects disease before other imaging tests. Magnetic resonance imaging (MRI) on the other hand is a technique which is used in radiology to create images of physiological processes and anatomy of a body. It uses radio waves and magnetic fields that help in generating the images of internal organs of human body.

Among all techniques, the CT scan is the most popular due to having the advantages of availability, rapid acquisition of the scan especially in lungs region, and cost [8]. The CT scan technique is most widely used in clinics for nodule identification and for its diagnostics. Unlike other techniques, the CT scan is very effective as it provides detail images in three dimensions of any tissue in human body and avoids the overlapping of layers of various tissues in images [9]. According to National Lung Screening Trial by Cancer Institute, the use of CT scan in detecting the nodule reduces the mortality of cancer by 20% in lungs in recent years. The research also shows the growth rate difference between radiologists and CT images. There is about 30% growth rate in CT images annually while the growth rate of radiologists are far less which is only 4, 1% annually so it is necessary to use this technique [10].

Patients having the symptoms of lungs cancer undergo CT scan in order to identify the abnormal growth in lungs region. After performing the CT scan of patient, the radiologist examines the reports to identify and detect the nodules that are suspicious from various CT scan images. These nodules are evaluated by the radiologists on the basis of chances of malignancy which is examined though given nodule information; the information may include morphology of nodule, its density, and texture feature.

After identifying the suspicious nodules, the next step is to perform the treatment but identifying these malignant nodules is a precise task. Due to radiologist distraction, inappropriate experience or fatigue in analyzing the scan may cause the probability of detecting and identifying the malignant nodules incorrectly with the available data. The probability of correctly detecting the nodules by radiologists is less than 52%. Moreover, a lot of time and effort is required by the radiologist in order to distinguish whether the nodule that is identified is malignant or benign, so human errors may occur in manual identification of abnormal nodules in lungs region.

To overcome this problem of human errors that occur during the detection of nodules in lungs, the framework is used that is computer-aided detection (CADe) framework. The CAD is basically pronounced as computer-aided detection (CADe) or computer-aided diagnosis (CADx). In computer-aided detection system, the analysis of number of images is performed by the system automatically [11]. It is a pattern recognition software that detects suspected abnormal features shape, texture, and growth rate from images and inform it to the radiologists in order to reduce the number of modules that may be missed by radiologist in other words it marks those areas of images that seems to be abnormal. It is designed to decrease the false positive rate and the observational sight of radiologist and aid him to evaluate the images more precisely. The CAD system, after analyzing the images, generate input for the radiologists to precisely identify the suspicious nodule from images generated through CT scans. Using the CAD not only provide valuable information but also reduces the workload. The important parts of CAD system include false positive reduction and nodule candidate detection [12]. It may be possible that the benign nodule may be misunderstood as malignant or malignant nodules may be interpreted as normal ones so reduction in false positive rate is required in order to eliminate the wrong finding from images while in the nodule candidate detection as many as possible nodules are to be identified from images.

The computer-aided detection workflow mainly consists of four major steps which include preprocessing, segmentation, feature extraction, and classification of nodules. In the preprocessing step, the raw data are organized and clean in order to remove noise from data and to prepare it for the next step. Segmentation on other hand is a process in which the image is divided into various multiple segments also called image objects or pixels. The main objective of segmentation process is to make the image more meaningful and authentic. In the process of feature extraction, we reduce the dimensionality so that the raw data are categorized into more manageable data in order for the further processing of data. The feature extraction is required when numbers of resources are required to be reduced for further processing without the loss of any relevant and important information. Classification is a process in which the given data are categorized into various classes. The process includes predicting the class which also refers as label, target, and category such as in the case of lung cancer the nodules are to be classified.

Since CAD frameworks, consists of already-defined stages that includes, division, prepreparing, classifications, and highlight extraction, in preparing different filters, for example, Gabor, Erosion, Median, Gaussian, and furthermore, various other methods can be utilized to get a sharp picture [13].

Convolutional neural network (CNN) is used for segmentation. Convolutional neural network is basically a deep learning algorithm which is designed in order to process arrays of data that include images also represented as (CNN/CovNet). The CNN require less processing as compare to other classifiers. The convolutional neural network reduces the image into such form that make it possible to process a large number of images easily without losing important features that are required in order to get a good prediction so CNN along with scalability of data also being effective in learning various features automatically and picking up deep patterns making it efficient for image processing. In CNN, instead of data preprocessing for extraction of feature the convolutional neural network get image pixel data automatically extract the features and also infer the objects that the image constitute. The CNNs are widely used in various areas such as object detection, image recognition, face detection, and image classification. The convolutional neural network takes an image as input process the image and classify it into various categories the system recognize image as an array of pixel that varies on image resolution [14]. In later phases, parameters, for example, geometric, factual, and HOG are derived utilizing linear discriminant analysis (LDA), free component analysis (ICA), and principal component analysis (PCA). Last phase is classification, where the recently-derived characteristics are categorized utilizing machine learning algorithms that include, fine KNN, Logit Boost, and SVM. In this research work, a novel segmentation method is proposed that is based on pixel-level segmentation and semantic segmentation for extraction of the region of interest (the affected area from the CT scan images of lung nodule detection). After extracting the affected area which is possibly benign and malignant will further than processed to extract the optimal features. The classification after feature extraction multiple classifiers like total boost, SVM, Logit boost medium KNN, and many more are tested, and the classifier with best results is implemented during the classification phase.

Our contribution includes precisely identifying the nodules from other lung structure by segmentation using CNN that achieve promising results compare to previous work. Multiple optimal features are selected to classify the nodules on the basis of their size, shape and other geometric, HOG, and LBP features. Eight different classifiers are trained and tested on these optimal features, and classification results are improved in the term of accuracy, sensitivity, precision, false positive rate, and error rate.

The key contributions of this paper include the following points:

- Precisely segmented nodules from other structure using CNN that achieved promising results compared to previous work
- Optimal features were selected to classify the nodules mainly on the basis of their geometric, histogram of oriented gradients (HOGs), and local binary patterns (LBPs) features.

The remaining article contains five major sections.  Section 2 explains previous research, while Section 3 delineates the proposed classification model and methods.  Section 4 discusses the results; Section 5 comparison with previous studies, whereas lastly in Section 6, the important points are out to the limitations and research gaps with overall conclusions and future directions.

## 2. Related Work

This section provides the summary of techniques commonly used by researchers for identification of lung cancer and their results. In a recent study, Ahmed et al. [15] tested 3D convolutional neural network on LUNA16 (lungs nodule analysis) dataset of 100 patients to identify the effected nodules. First, preprocessing was performed using thresholding technique, which itself contained two stages, i.e., resizing the image and averaging it. The substance: air and other noises were removed during segmentation. Vanilla 3D CNN was used for classification of both noncancerous and cancerous images and achieved the accuracy of 80%. Cao et al. [16] proposed multibranch ensemble learning architecture (MBEL) with 3D CNN that contains three network models (ResNet, DenseNet, and VGGNet). The results were based on the average of each model output probability. Around 87% accuracy was achieved on the LUNA16 dataset. To assist the radiologist in exactly identifying the location of the nodule from images, Xie et al. [17] proposed a method which at first detected the nodule candidate by adjusting the R-CNN with deconvolution. Then, 2D convolutional neural network (2D-CNN) was used to evaluate true nodule candidates. This research also used the LUNA16 dataset achieving overall sensitivity of 86.42%. Cao et al. [18] developed a method containing two stages; the first stage uses U-Net for nodule candidate detection by just segmenting the malignant ones. The second stage is focused on false positive rate reduction through dual pooling structure with 3D CNN. It demonstrated an accuracy of almost 93%. Liu et al. [19] proposed a network structure-based method using Inception Net-based 3D model, ResNet-based, and VGGNet-based network architecture. By combining the output of these architectures, the author achieved good final results.

Two convolutional neural networks were also proposed by [20] to detect the lungs nodule accurately. Their proposed method contains two CNN models. The first one includes a hybrid 3D CNN with RBF-SVM (radial basis function-support vector machine) and the second comprised a straight 3D CNN with SoftMax, which achieved an accuracy of 91%. Another model employing 3D deep CNN with computer-aided detection (CAD) technique and multiscale prediction strategy was suggested by the authors in [21]. It consists of two major steps: first one is lungs segmentation and other is nodule candidate detection. The segmentation step used the threshold method to extract the accurate lungs nodule region. They tested it on the LUNA16 dataset getting 89% of accuracy. Song et al. [22] proposed multiple deep learning methods that include deep neural network, convolutional neural network, and stack auto encoder classifier. The author applied these classifiers on computed tomography images with modifications. The convolutional neural network contains multiple layers such as pooling layers and convolutional layers, but the author also added softmax layer in CNN. Similarly, other two architectures were also implemented by the author to classify the lungs nodules. This study also trained and tested on the LIDC dataset.

Wang [23] proposed a MV-DCNN (a multiview deep convolutional neural network) for segmentation of nodules. The special feature of multiview deep convolutional network was that its ability to capture various sets of sensitive features of nodules by providing three different views of computed tomography images. The overall performance achieved by this model was 77.58%. The authors in [24] discussed about transfer learning-based guided approach in assistance of recognition models where it is being guided via domain generation in intermediate of networks.

Rodrigues et al. [25] proposed using structural cooccurrence matrix- (SCM-) based method to identify malignant nodules and classify their malignancy levels. The classification stage used: multilayer perceptron (MLP), SVM, and KNN classifiers, achieving 74.5% accuracy. Woźniak et al. [26] used local variance analysis and probabilistic neural network (PNN) to achieve 92% of correct predictions, when classifying lung carcinomas. Filho et al. [27] adopted spatial interdependence matrix (SIM) and visual information fidelity (VIF) combined with the optimum-path forest (OPF) classifier to recognize the lungs as normal or affected with fibrosis. Ke et al. [28] suggested using a neural network combined with heuristic Moth-Flame and Ant Lion algorithms to recognize degenerated lung tissues in X-ray images. Capizzi et al. [29] employed type-1 fuzzy rules combined with a neural network to recognize lung nodules with an accuracy of 92.56%. Chouhan et al. [30] suggested an ensemble model that combines outputs from pretrained neural network models for pneumonia recognition and got an accuracy of 96.4%. Nóbrega et al. [31] employed deep feature extractor based on the ResNet50 and the SVM RBF classifier, achieving an accuracy of 88.41% for early-stage lung cancer recognition. Khan et al. [32] used multiple texture, point, and geometric features, which were fused using correlation-based fusion. Most discriminate features were used with ensemble classifier, achieving an accuracy of 99.4% for lung cancer recognition.

Sahlol Elaziz et al. [33] combined a pretrained MobileNet network model with artificial ecosystem-based optimization (AEO) algorithm as a feature selector to achieve 90.2%–94.1% accuracy for tuberculosis recognition. Sahlol Yousri et al. [34] used Inception CNN to extract features and a marine predators algorithm to select the most relevant features for COVID-19 X-ray classification, achieving very high performance. Souza et al. [35] performed lung segmentation using Mask R-CNN model to create a pulmonary map and applied fine-tuning to find the pulmonary borders on the CT image with an accuracy of 98.34%. Khan and Hussain et al. [36] used pretrained DenseNet-201 network for feature extraction, and a Firefly algorithm to select the best learning features. Fused features were classified using an extreme learning machine (ELM), achieving an accuracy of 94.76% for classifying COVID-19 CT scans. Khan and Kadry et al. [37] developed a custom 15-layered CNN architecture to extracts deep features from chest CT scans images. Deep features were combined using the max-layer detail (MLD) method and classified with one-class kernel ELM classifier to reach an average accuracy of 95.1%.

All the abovementioned techniques have been quite successful in contributing for lung cancer nodule detection, but the studies still need satisfactory results due to challenges regarding heterogeneity of lungs nodules in terms of their shape, size, and texture. The nodules have certain location and morphology as well, so they also need to be considered. Radiologists also need to reduce the diagnosis time of CT scans as per slice takes 2 to 3.5 minutes to diagnose them manually [38].

## 3. Materials and Methods

**Figure 1:**
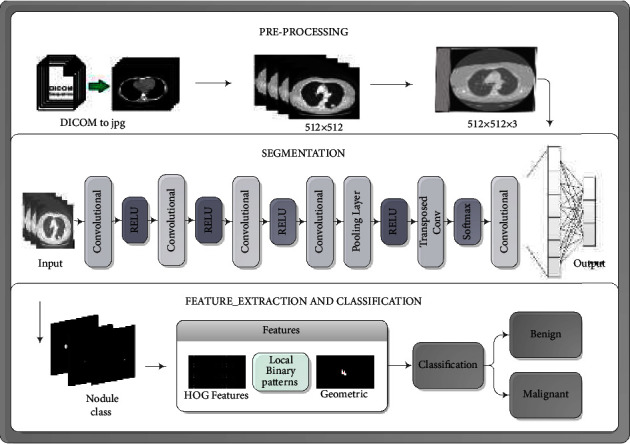
Workflow of the proposed methodology.

Proposed framework (Figure 1) has multiple stages that include preprocessing, segmentation, feature extraction, and classification; each discussed in the next sections. LIDC image slices are used in which there are 146 images are firstly converted from DICOM to jpg format. The method begins with data preprocessing to get the data into normalized form. After format conversion, ground truth labels mapped to create labeled images, segmentation is performed in which background, and other unnecessary parts are separated from nodules. As nodules have certain shape, location, and size for both malignant and benign, we extract multiple features from segmented nodules. These features are geometric, HOG and LBP features, discussed in the next sections. Based on these features, classification is performed to distinguish the malignant and benign nodules. Classifiers are trained and then tested with 10-crossfold validation method. The fusion of features is evaluated in terms of time of training of classifiers and prediction accuracy.

Methodology of a proposed framework is shown above in Figure 1 which includes three phases the first phase is pre-processing in which normalization of data is performed and noise is removed. The next phase is segmentation in which CNN is used to separate the region of interest and third phase is classification that classifies the nodules into two main classes based on multiple features.

### 3.1. Preprocessing

Since both the machine and deep learning approaches require huge amount of data to provide a solid foundation in the form of reliable patterns for learning as well as to further process the data, the data are labeled with an intent to assist the model in detection of discriminative, informative, and independent patterns. Labeling is performed according to the given ground true values in the dataset. These ground truth values indicate annotated or marked-up features that needs to be segmented by the CNN as nodule class. The labeling images with nodule and background class are created which is later given to CNN with input images as input data.

### 3.2. Segmentation

Many of the recent studies have used deep learning for segmentation [39] where like this inspiration, the proposed study also used DL for segmentation purpose. Similar to this, DL used in various recognition and classification tasks now-a-days [40]. To process the key elements of images, rather than doing the whole image, segmentation is performed in the region of interest (ROI) separated from others. In this way, relevant features or depicted objects that further assist in the classification process are obtained. The segmentation is performed for purpose of separating the lungs nodules from other complex background structures. A performance based selected custom fourteen-layer CNN is proposed in which images of dimension 512 × 512 × 3 is input to the CNN having zero center normalization. The second layer of proposed CNN is convolutional layer, where the kernel/filters of dimension 3 × 3 is applied to the input data. In these convolutional operations, 3 × 3 kernel filter slides over each input image by setting stride (no. of pixel shifts in a matrix) of [1 1] to 32.

Later on, the transposed convolutional layer is added to carry the trainable up-samples if any. This process is repeated for the whole image and output channels are generated as a result. The details of CNN layers are covered in Table 1.

(1) $$\begin{array}{c} G\left(m,n\right)=\underset{j}{\sum }\underset{k}{\sum }h\left[j,k\right]f\left[m-j,n-k\right], \end{array}$$

where “*f*” represents the input image passed onto convolutional layer and “*h*” represents the kernel that has been applied to the image. “*m*” and “*n*” represent the rows and columns of the image and “*j*” and “*k*” represent the filtered rows and columns, respectively. An activation function called rectified linear unit (ReLU) is then applied to the feature map generated by the convolutional layer that returns a “0” in response to negative input values. Again, the convolution layer is applied to the previous layer output performing the convolutional operation with 3 × 3 filters and stride of [1 1] and padding of [1 1 1 1] mainly to get deeper pixels. Rectified linear unit is also applied on previous feature map to remove the negative values. After getting the output channel, it is then passed to the next convolutional layer that again applies 3 × 3 size filters on the input channels with same stride and padding. These all operations ultimately removing the nonlinearity from input channel and forward it to next layer. A max pooling filter of a dimension 2 × 2 is then applied to the input of previous layer for extraction of the most common features in images and reducing the spatial volume of images. In order to decrease computational expenses a stride of [2 2] and padding of [0 0 0 0] is applied in this layer.

(2) $$\begin{array}{c} h_{xy}^{l}=\underset{i=0\ldots ,s,j=0\ldots ,s}{\max}h_{\left(x+i\right)\left(y+j\right)}^{l-1}. \end{array}$$

Max pooling operation returns the max value in each patch and stores it as output. This process is then repeated for the whole image and max pixel value from each patch is then returned.

The next layer is the same convolution layer with 3 × 3 filters along with the same stride and padding used in above convolution layers in addition to the ReLU activation function to remove nonlinearity. The next layer is a transposed convolution layer that is used for up sampling. The filter used here are 4 × 4 dimension with a stride of [2 2] and cropping of [1 1 1 1] in this layer. Another layer of convolution is then applied on the feature map generated by the transpose layer using 1 × 1 filter with the same stride [1 1], but a different padding of [0 0 0 0]. The SoftMax activation function is then applied to the output generated in the convolution layer. This function predicts probability of segmentation classes.

(3) $$\begin{array}{c} {\left(Z\right)}_{i}=\frac{e^{z_{i}}}{\sum_{j=1}^{k}e^{z_{j}}}\text{.} \end{array}$$

**Figure 2:**
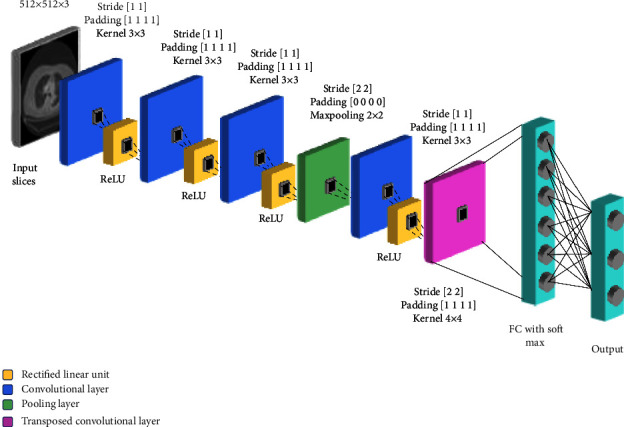
Proposed convolutional neural network for nodule segmentation.

Here, “(*Z*)_*i*_” represents the input values and “*e*^*z*_*i*_^” represents the exponential of input values which is divided by the sum of exponential values, where “*k*” represents the number of multi classes whose probability is to be predicted. Finally, the pixel classification layer is applied. The proposed architecture of CNN is depicted in Figure 2.

### 3.3. Feature Extraction

To reduce the number of resources for further processing and without losing important information, multiple features are extracted in this phase but only those features are used to classify the nodules that produce effective results all other noisy features (features from which we attain less accurate results) are separated in this article; three different features are used that assist us in classification. These features are histogram of oriented gradients (HOGs), local binary pattern (LBP) features, and geometric features.

### 3.4. Histogram Oriented Gradients (HOGs)

By influencing, the HOG features as discussed in [41], the proposed study uses it for objects detection in images. As object detection is one of the main functionalities of the histogram oriented gradient features. The HOG divides each image into small portion or cells it then computes the histogram of oriented gradient of every cell of images then using block wise pattern to normalize the cell. First step is to resize the image to standard dimension, and then gradient of each cell is calculated. Since in proposed study, 4 × 4 size of patches in an image is used. Each patch contains multiple cells having pixel values from which a new matrix is generated. In order to calculate the gradient in *x* direction, each right pixel value from central pixel is taken and subtract from the value on its left similarly. In case of *y*-axis direction, each upper pixel value from central pixel is subtracted from the pixel value which is below to it. After getting this operations, two new matrices of a patch are generated which contain storing gradients of both *x* and *y* directions. Next, the magnitude and direction of each pixel is calculated and a histogram is generated from these orientations and directions.

(4) $$\begin{array}{c} Magnitude=\sqrt{{\left(G_{x}\right)}^{2}+{\left(G_{y}\right)}^{2}}. \end{array}$$

Equation (5) is used to calculate the magnitude where “*G*_*x*_” represents the gradient along *x* direction and “*G*_*y*_” represents the gradient along *y* direction.”

(5) $$\begin{array}{c} \emptyset =\mathrm{atan}\;\left(\frac{G_{x}}{G_{y}}\right). \end{array}$$

**Figure 3:**
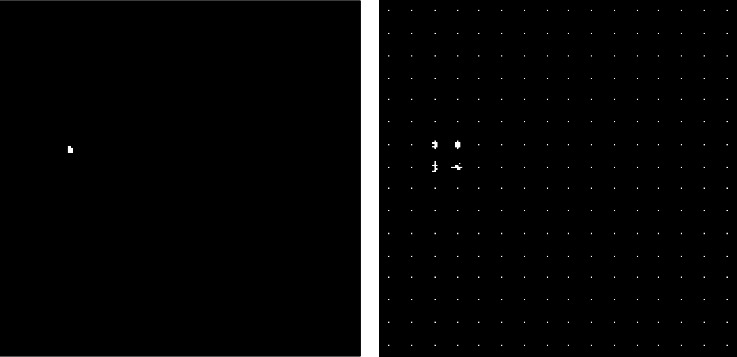
Visualization of extracted hog features. (a) Benign case. (b) Extracted hog features.

Same process is repeated for every pixel in the image and histogram is generated for every image as shown in Figure 3.

### 3.5. Local Binary Pattern (LBP)

As both classes have distinguishing features so by inspiring from the work of [42] the specific pattern of both classes are extracted. For every pixel in the image, a fixed size of 4 × 4 neighborhood is selected. Then, a pixel is selected in a matrix and threshold is checked against its each neighbor. Value “1” is assigned in the output matrix if the pixel intensity is equal or greater than the neighbor pixel otherwise “0” is assigned to the neighbor's greater than the selected pixel. LBP of every pixel is calculated by storing binary values in an array in clockwise direction. The binary value for output matrix is then converted into a decimal number that represents the output pixel value.

(6) $$\begin{array}{c} \overset{7}{\underset{n=0}{\sum }}s\left(\iota_{n}-\iota_{c}\right)2^{n}. \end{array}$$

Here, *n* represents the number of iteration while 7 is the total number of neighboring pixels around the central pixel. *ι*_*n*_ represents the neighbor pixel which is subtracted from central pixel represented as *ι*_*c*_.

(7) $$\begin{array}{c} S\left(z\right)=\left\{\begin{array}{cc} 1, & z\geq 0,\\ 0, & z\leq 0. \end{array}\right. \end{array}$$

Equation (7) assigns a “1” in the output matrix if the output value of the operation performed between neighbor and central value is equal to or greater than the central pixel value, otherwise it is assigned “0.” This process is repeated for every pixel in the image and LBP features are extracted this way.

### 3.6. Geometric Features

**Figure 4:**
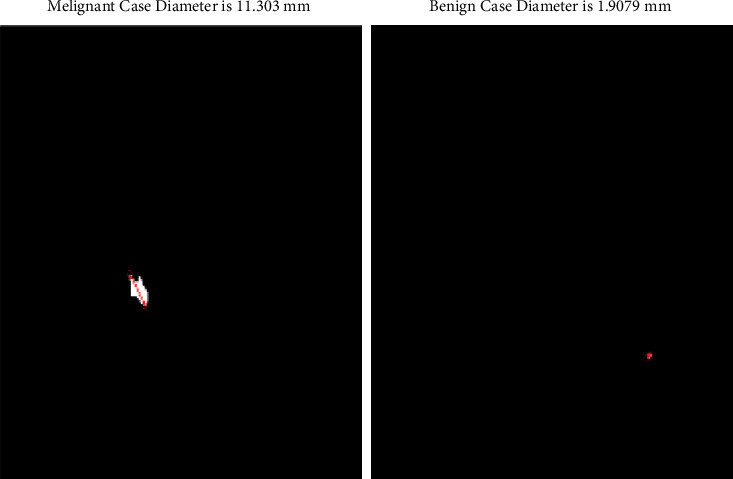
A visualization of some of the extracted geometric features.

Another type of features that are extracted from the images are geometric features. These features extracted from segmented lesions as diameter of polygons. The visualization of the diameter of the nodules is shown in Figure 4. Another study has used size as features to classify the nodules. By inspiring that study, the proposed study has used convex hull to get maximum distance points and then later on used Euclidean distance to measure distance between calculated points [43]. This feature plays a vital role in the classification of malignant and benign nodules. Euclidean distance is calculated for the diameter from every perspective of nodules which typically finds the maximum distance between the points. The maximum distance between two points is considered as the nodule diameter because of the existing having irregularity in its shape.

### 3.7. Features Fusion

First, the HOG features are extracted and passed to different machine learning classifiers, secondly HOG and LBP features are fused using concatenation method and then classified the lungs nodules, where lastly the HOG, LBP, and Geometric feature all concatenated and classified using different machine leaning classifiers with their prediction accuracy and training time. All features classification results are discussed in later section.

### 3.8. Classification

On the basis of features extracted from images, the classification is performed in order to differentiate the malignant nodules from benign. Multiple classifiers are applied to test and seek the most effective classification results: Bagged ensemble, subspace discriminant, subspace KNN, RUSBoost, fine, medium, and coarse tree, linear, quadratic, cubic, medium Gaussian support vector machines (SVMs), fine, medium, coarse, cosine, cubic, and weighted KNN.

## 4. Results and Discussion

### 4.1. Running Environment

All the experiments were performed on core i7 5th generation octal-core system with 32 GB DDR 4 RAM with a bus speed of 2400 MHz along with a dedicated graphic processing unit (GPU) of 8 GB memory. Windows 10 64-bit operating system is installed on this computer. To detect the lungs nodule and to classify them as malignant and benign, both the training and testing is performed using MATLAB. All the experiments including pre-processing of dataset images, segmentation of nodules along with feature extraction and implementation of multiple classifiers and results are generated also using MATLAB.

### 4.2. Dataset Description

The lungs image database consortium (LIDC) dataset is used in our classification framework for the detection of lungs cancer [44]. The LIDC contains lungs cancer screening CT scans. A specific number of relevant slices are taken according to DICOM standards. The slices are available in 512 × 512 dimensions. The header in these DICOM files contains information about slice parameters such as spacing, pixels, and thickness. LIDC contain 1018 overall patient cases. About 146 CT slices were taken according to DICOM standards for testing and training purpose. These slices are two-dimensional images that are converted into three dimensional slices of 512 × 512 × 3. Information about CT scans is available in XML files which are publicly available on cancer image archive [44]. The detail properties of dataset are given in Table 2.

#### 4.2.1. Experiment Results

The proposed framework used convolutional neural network is used for the process of segmentation. By using CNN for segmentation, the nodules are distinguished from other background structures of lungs. The CNN model is trained on 70% of images and tested on the rest 30%. The overall performance achieved in segmentation is shown in Table 3.

These nodule images are then separated, and nodule features are extracted from these images. Subsequently, classification is performed to distinguish the cancer nodules from benign (normal) ones, using multiple classifiers.

### 4.3. Classification Using LBP Features

After getting segmented nodules, the proposed study extracted LBP features using 4×4 neighborhood operation as discussed earlier. After getting features, 17 different classifiers are used with 10-fold cross validation. Their predicting accuracy with their training time and prediction speed is given in Table 4. Time complexity of cubic KNN is lowest among all of the classifiers which also achieves same accuracy level as compare to worst time taker classifier. Moreover, the best prediction accuracy achieves by subspace KNN with 93.1% using 34.081 sec which can be considered as best model among all classifiers as compare to time complexity and accuracy level.

### 4.4. Classification Using HOG-LBP Features

The extracted LBP features later are concatenated with HOG features and validated on same classification models using 10-fold cross validation method. The results are given below in Table 5. By concatenating two types of features, we observe a reduction in accuracy level but as compared to Table 4, the training time reduce to a maximum time of 38.98 sec which is approximately equal to 39 sec. We can observe that time complexity reduces using LBP and HOG features where the accuracy also reduces, which is not so promising to get higher results to identify the malignant and benign nodules.

### 4.5. Classification Using HOG-LBP-Size Features

All features of HOG, LBP, and size of nodules are concatenated in parallel and given to the same classifier using the same validation method. The results obtained through LBP-HOG are presented in Table 6.

**Figure 5:**
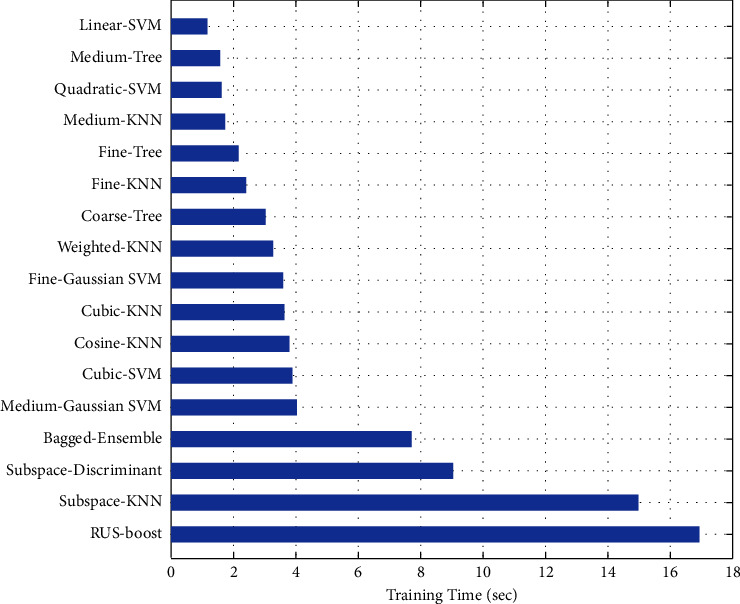
Training time of classifiers using LBP-HOG-Size features.

**Figure 6:**
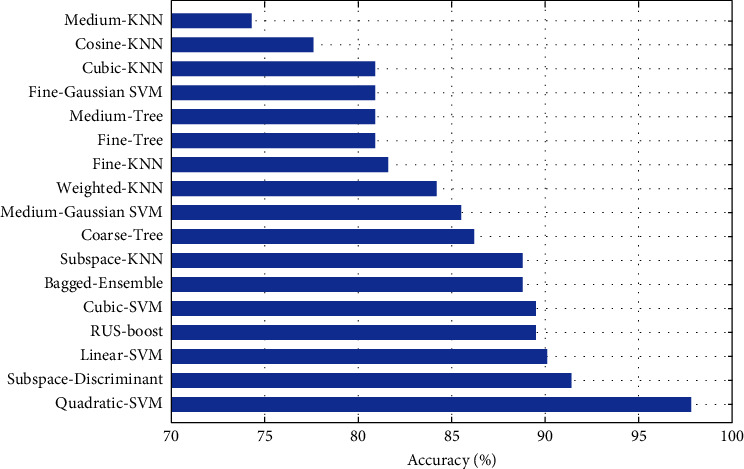
Accuracy comparison of classifiers using LBP-HOG-Size features.

By including size or geometric features into features vector, we got a high accuracy result in case of quadratic SVM with lower time consumption of only 1.161 sec., which is considerable as a classifier for malignant and benign nodules. For all classifiers, time of training and accuracies are shown in Figures 5 and 6.

The proposed approach of setting optimal features with performance measure of training time and accuracy clearly reduce the time complexity and increase the accuracy rate. The maximum training time reduce from more than one minute to 16.93 sec where the most accurate results of quadratic SVM achieve the accuracy of 97.83% in much lower time of 1.161 sec.

To compare which proposed study best achieves results with state-of-the-art approaches, a comparison is shown in Table 7. By observing accuracies, sensitivities and specificity rates of previous studies as compared to proposed study the achieved accuracy is 97.83% with 93.3 specificity and 100% as sensitivity. The sensitivity is proportion of total number of effected cases that are correctly predicted by the models over total number of actual cases The sensitivity of all the classifiers is then calculated in which the support vector machine achieves the sensitivity of 1.0 while other classifiers that include fine KNN, Gentle boost, Logit boost, Robust boost, medium KNN, Total boost, and subspace achieve sensitivity of 0.87, 0.91, 0.87, 0.87, 0.73, 0.8, and 1, respectively.

Moreover, the time complexity in proposed study as discussed in previous section is much lower which makes the proposed study as more optimal approach to classify the lungs nodules. The pseudocode of the employed framework is presented in Algorithm 1.

An accurate segmentation of cancer nodule in lobe region is one of the most challenging tasks. There exists some trachea and vessels in the lobes region which are difficult to differentiate it from nodules. Due to same intensity of these vessels and bronchi in lobe regions of lungs, correct identification and extraction of ROI with same intensity is very complex. Many previous approaches as mentioned in related work did not use combination of both automated features and handcrafted features they either identify the nodules using only handcrafted features or just through automatic feature extraction using deep learning. Some approaches as mentioned in related work section obtained results through both machine and deep learning but unable to use the best combination of features that result in lower accuracy of their models. We tested out dataset on various set of features and selected those that could result in better accuracy and sensitivity.

## 5. Comparison

To prove the validity of work, the proposed is compared with recently published studies on lungs focal opacity identification. The comparison is shown in Table 8. The first study is published in 2019 and has shown 93.2% accuracy with 91.3% specificity and 93.1 sensitivity rates.

The second comparison that proposed in 2020 has shown 94% accuracy, 93.9% specificity, and 83.7% sensitivity, and the last one is proposed in 2021. It has shown 96.33% that is more than the previous studies with 96.37% sensitivity which is more than the previous studies as well. However, the proposed study that is conducted in 2022 and outperformed better with 97.8% accuracy, specificity, and sensitivity.

## 6. Conclusion

To detect tumor art early stage, we proposed a framework that assist the radiologist in identifying the malignancy of nodules so that patients can be diagnose form this deadly disease. The framework contains mainly the segmentation and classification of nodules. The segmentation is performed using proposed convolutional neural network contain multiple hidden layers. The segmented slices are then classified using features to identify the effected nodules that will help the radiologists using computed tomography images.

Results proved that our framework achieves better results in both detection and classification of lungs nodule as compared to other state-of-the-art approaches. It is also noticed that the features selection and classification also need to be considered as it reduces the overall training time and prediction speed with higher accuracy results. In future, researchers can use the optimal features selection based upon less time complexity to get higher accuracy results. It is suggested to use more optimal selection of features and more dataset images to get more promising results.

Reinforcement learning (RL) is paradigms of machine learning that learns by having an interaction with environment. Reinforcement learning can learn from consequence of action, feedbacks, and past experience rather than being taught to make useful decisions. So RL can be used in future for detecting the lung cancer to achieve better results as the RL has great ability to achieve its goals in potentially complex and uncertain environments.

Lung's cancer has multiple stages that include from stage 0 to stage 4 cancers. Stage 0 includes presence of a small tumor which can either be benign or malignant in lung. First stage includes presence of cancer in lung tissues. In second stage cancer spread to lymph nodes while in third stage it spreads into other organs like chest and last stage it may spread in whole body. So identifying the lungs cancer stages is necessary for proper diagnosis of diseases. In future, multiple stages of lungs cancer will need to be identified accurately to assist radiologists in proper diagnosis of disease. Furthermore, our framework will be trained and tested on other datasets to enhance the performance.

## Figures and Tables

**Algorithm 1:**
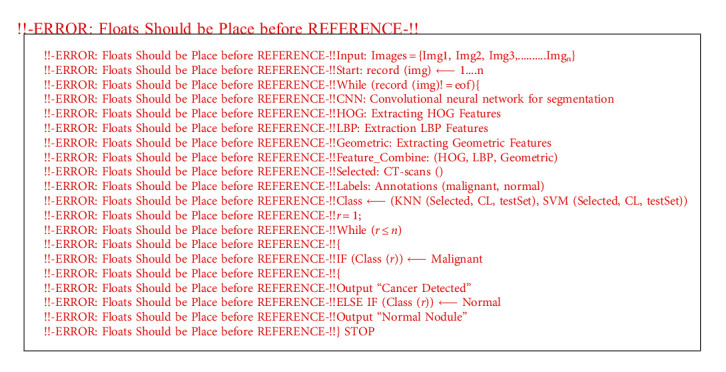
Pseudocode for the proposed system.

**Table 1:** Description of the convolutional neural network layers.

Layers	Filters	Activation	Filtersize	Stride	Padding
Convolution	32	ReLU	3 × 3	[1 1]	[1 1 1 1]
Convolution	32	ReLU	3 × 3	[1 1]	[1 1 1 1]
Convolution	32	ReLU	3 × 3	[1 1]	[1 1 1 1]
Max pooling			2 × 2	[2 2]	[0 0 0 0]
Convolution	32	ReLU	3 × 3	[1 1]	[1 1 1 1]
Transposed convolution	32		4 × 4	[2 2]	[1 1 1 1]
Convolution	2	Softmax	1 × 1	[1 1]	[0 0 0 0]
Pixel classification layer					

**Table 2:** Properties of dataset used in experiments.

Properties	Description
Format	DICOM
Size	512 × 512 × 3 resolution
Dataset	LIDC
Number of images	146
Number of nodules	152
Benign	103
Malignant	49
Image type	CT scans

**Table 3:** Overall segmentation results.

Global accuracy	Mean accuracy	Mean IoU	Weighted IoU	Mean BF score
0.95197	0.88162	0.66145	0.92441	0.7297

**Table 4:** LBP Feature based classification results.

Methods	Prediction speed (obs/sec)	Training time (sec)	Accuracy (%)
Bagged ensemble	240	26.813	90.8
Subspace discriminant	140	34.813	87.5
Subspace KNN	110	34.081	93.4
RUSBoost	320	37.822	84.2
Fine tree	610	39.573	84.2
Medium tree	640	30.62	84.2
Coarse tree	3300	66.487	86.2
Linear SVM	1200	11.318	90.8
Quadratic SVM	1300	9.416	92.1
Cubic SVM	2500	11.899	92.1
Fine Gaussian SVM	2600	11.64	90.8
Medium Gaussian SVM	2400	11.51	92.1
Fine KNN	540	6.581	92.1
Medium KNN	740	4.525	87.5
Cubic KNN	820	8.64	86.2
Cosine KNN	910	8.94	91.4
Weighted KNN	1900	7.164	91.4

**Table 5:** LBP-HOG features based classification results.

Methods	Prediction speed (obs/sec)	Training time (sec)	Accuracy (%)
Bagged ensemble	260	38.98	88.2
Subspace discriminant	140	15.307	90.1
Subspace KNN	110	11.841	78.9
RUSBoost	2800	22.179	87.5
Fine tree	740	2.997	82.2
Medium tree	760	2.155	82.2
Coarse tree	1000	7.058	85.5
Linear SVM	520	5.95	86.8
Quadratic SVM	520	4.90	88.8
Cubic SVM	690	6.77	87.5
Fine Gaussian SVM	780	6.59	82.2
Medium Gaussian SVM	700	7.503	83.6
Fine KNN	560	3.87	80.9
Medium KNN	610	2.11	71.1
Cubic KNN	280	4.81	76.3
Cosine KNN	600	4.49	79.6
Weighted KNN	620	4.699	82.9

**Table 6:** LBP-HOG-Size features-based classification results.

Methods	Prediction speed (obs/sec)	Training time (sec)	Accuracy (%)
Bagged ensemble	220	7.71	88.8
Subspace discriminant	160	9.043	91.4
Subspace KNN	120	14.98	88.8
RUSBoost	360	16.93	89.5
Fine tree	710	2.16	80.9
Medium tree	790	1.57	80.9
Coarse tree	1000	3.03	86.2
Linear SVM	560	1.161	90.1
Quadratic SVM	620	1.618	97.8
Cubic SVM	550	3.79	89.5
Fine Gaussian SVM	760	3.59	80.9
Medium Gaussian SVM	750	4.03	85.5
Fine KNN	680	2.4	81.6
Medium KNN	570	1.73	74.3
Cubic KNN	630	3.63	80.9
Cosine KNN	340	3.73	77.6
Weighted KNN	630	3.27	84.2

**Table 7:** Summary of recent state-of-the-art approaches.

Serial no.	Ref	Title	Methods	Accuracy (%)	Specificity (%)	Sensitivity (%)
1	[22]	Using deep learning for classification of lung nodules on computed tomography images	CNN with deep neural network and stack auto encoder	84.1	83.9	84.3
2	[45]	Lung's nodule classification using combination of CNN, second, and higher order features	CNN withharalick, grey level run length matrix and spatial features	93.5	86.6	96.5
3	[46]	A computer-aided pipeline for automatic lung cancer classification on computed tomography scans	LUVEM with energy shape and texture features	96	97.4	94.2
4	[47]	Lung nodule detection in CT images using statistical features	Histogram based threshold technique with statistical and shape features	92	91	93.9
5	[48]	Artificial neural network-based classification of lung nodules in CT images using intensity, shape, and texture features	ANN with texture, shape and intensity features	93.2	91.3	93.1
6	[19]	Multimodel ensemble learning architecture based on CNN for lung nodule malignancy suspiciousness classification	CNN-based multimodal framework (VGGNet, InsepNet, ResNet)	94	93.9	83.7
7	[49]	A machine learning approach to diagnosing lung and colon cancer using a deep learning-based classification framework	DL-based convolutional neural network	96.33%	NA	96.37
8	Proposed	Identification and classification of lungs focal opacity using CNN segmentation and optimal feature selection	CCN with geometric, HOG, LBP features and SVM classifier	97.8	93.3	100

**Table 8:** Comparison with state-of-the-art studies.

Serial no.	Ref	Title	Methods	Accuracy (%)	Specificity (%)	Sensitivity (%)
1	[48]	Artificial neural network-based classification of lung nodules in CT images using intensity, shape, and texture features	ANN with texture, shape and intensity features	93.2	91.3	93.1
2	[16]	Multimodel ensemble learning architecture based on CNN for lung nodule malignancy suspiciousness classification	CNN-based multimodal framework (VGGNet, InsepNet, ResNet)	94	93.9	83.7
3	[49]	A machine learning approach to diagnosing lung and colon cancer using a deep learning-based classification framework	DL-based convolutional neural network	96.33%	NA	96.37
4	Proposed	Identification and classification of lungs focal opacity using CNN segmentation and optimal feature selection	CCN with geometric, HOG, LBP features and SVM classifier	97.8	93.3	100

## Data Availability

The data are publicly available where further details are included within the article. Code and data are available from the corresponding author upon reasonable request.
